# A nomogram for predicting the risk of coronary artery disease in premenopausal women with suspected coronary artery disease

**DOI:** 10.1038/s41598-025-14589-6

**Published:** 2025-08-11

**Authors:** Yahui Qiu, Qifeng Guo, Xuejuan Feng, Weiqiang Xiao, Shisen Liang, Mei Wei

**Affiliations:** 1 Department of Heart Center, The First Hospital of Hebei Medicical University, 89Donggang Road, Yuhua District, Shijiazhuang, 050000 Hebei China; 2 Graduate School of Hebei Medicical University, 361 Zhongshan East Road, Shijiazhuang, 050000 Hebei China; 3 Department of Nephrology, The First Hospital of Hebei Medicical University, 89Donggang Road, Shijiazhuang, 050000 Hebei China

**Keywords:** Premenopausal women, Coronary artery disease (CAD), Nomogram, Prediction model, Cardiology, Diseases, Risk factors

## Abstract

Due to the cardioprotective effects of estrogen, premenopausal women have a relatively lower risk of developing coronary artery disease (CAD). However, the incidence of CAD in premenopausal women has been increasing in recent years. Therefore, the aim of this study is to develop a clinical prediction model to estimate the risk of CAD in premenopausal women. This study included premenopausal women who underwent coronary angiography at the First Hospital of Hebei Medical University from September 2018 to December 2021. The Least Absolute Shrinkage and Selection Operator (LASSO) regression method was used to identify the optimal variables for predicting the risk of CAD in premenopausal women. A nomogram was then constructed using multivariate logistic regression analysis. Finally, the predictive performance of the nomogram was evaluated using the area under the receiver operating characteristic curve (AUROC), its calibration performance was assessed using calibration curves, and clinical net benefit was evaluated using Decision Curve Analysis (DCA). A total of 222 premenopausal women were ultimately included for analysis, of whom 86 were diagnosed with CAD. Through LASSO and multivariate logistic regression, five predictive variables were finally selected: age, diabetes mellitus (DM), aspartate transaminase (AST), alkaline phosphatase (ALP), and lipoprotein (a) (Lp(a)). These five variables were used to construct a prediction model, which was presented in the form of a nomogram. The calibration curves of the nomogram showed good fit. The area under the receiver operating characteristic curve (AUROC) for the nomogram was 0.819 (95%CI: 0.760–0.878). Additionally, decision curve analysis (DCA) indicated that the nomogram can achieve good net benefit in clinical applications.

## Background

It is well-known that coronary artery disease (CAD) is one of the leading causes of death and disability worldwide^1,2^. While traditionally considered more prevalent in men, the incidence of CAD in women has risen significantly over the past few decades. Women diagnosed with CAD often face higher morbidity and mortality rates compared to men: 38% of women who experience a heart attack die within one year, compared to 25% of men; 46% of women develop heart failure within six years after a heart attack, compared to 22% of men^3,4^. For premenopausal women, a specific subgroup, the protective effects of estrogen on the heart typically result in a lower incidence of CAD compared to other populations^5–7^. However, recent trends show an increasing incidence of CAD in premenopausal women^8^. Most women with CAD exhibit atypical clinical symptoms. Studies have shown that 49.6% of premenopausal women presenting with chest discomfort are diagnosed with CAD through coronary angiography, contradicting the widespread belief that the likelihood of CAD in this group is minimal. Therefore, it is crucial to carefully evaluate and diagnose chest discomfort in premenopausal women, as any oversight can have severe consequences. Currently, most scoring systems used to predict CAD are developed based on European and American populations, whose disease characteristics differ from those of Chinese populations. Additionally, these scoring systems are not specifically tailored for premenopausal women^38–40^. Thus, the aim of this study is to develop a clinical model using common indicators to predict CAD in premenopausal women. This model will help clinicians better assess the risk of CAD in this group and promptly implement appropriate treatment measures to improve patient outcomes.

## Materials and methods

### Study population

**Fig. 1:**
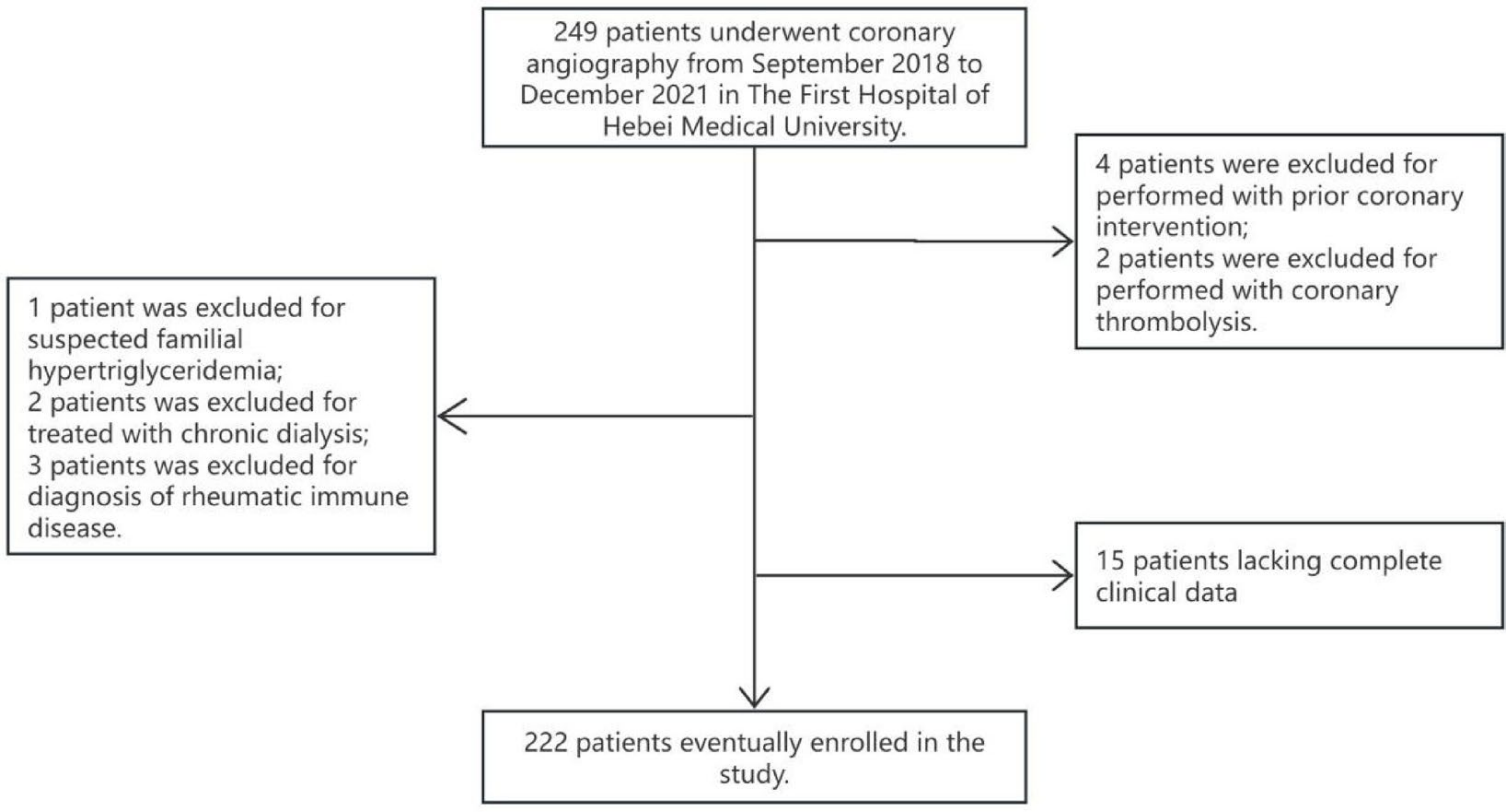
Flowchart of the study population enrollment.

A total of 249 premenopausal female patients underwent coronary angiography from September 2018 to December 2021 in The First Hospital of Hebei Medical University were enrolled. This group of people has at least one of the following characteristics: chest pain, chest tightness, shortness of breath, which is usually caused by fatigue, emotional stress, cold weather, or heavy meals. Chest pain is located anywhere between the sternum, upper abdomen, mandible, or in any arm. Discomfort usually manifests as tightness, heaviness, squeezing, or pressure, and may be accompanied by related symptoms including palpitations, nausea, or sweating. The above symptoms are relieved by rest or sublingual nitroglycerin. The menopausal status was defined as absence of menses for at least 12 consecutive months^9^. Major exclusion criteria were as follows: (1) Prior coronary intervention or coronary thrombolysis or coronary artery bypass grafting was performed; (2) Long-term use of drugs that interfere with normal estrogen secretion; (3) Chronic dialysis; (4) Severe hepatic dysfunction; (5) Severe acute infection; (6) Malignant tumor; (7) Suspected familial hypertriglyceridemia [plasma triglyceride(TG) ≥ 500 mg/dL (5.65 mmol/L) or more than one first-degree relative with TG ≥ 500 mg/dL]; (8) Rheumatic immune disease; (9) Severe psychiatric disorders; (10) Incomplete clinical data. According to the exclusion criteria, 4 patients were excluded for performed with prior coronary intervention; 2 patients were excluded for performed with coronary thrombolysis; 1 patient was excluded for suspected familial hypertriglyceridemia; 2 patients was excluded for treated with chronic dialysis; 3 patients was excluded for diagnosis of rheumatic immune disease; 15 patients lacking complete clinical data, and the detailed population screening process is shown in Fig. 1. Ultimately, a total of 222 patients participated in the study.

### Data collection and definitions

The patients’general clinical data such as age, height, weight, systolic blood pressure(SBP), diastolic blood pressure (DBP), low-density lipoprotein (LDL), high-density lipoprotein cholesterol (HDL), total cholesterol (TC), Triglycerides(TG), Uric Acid (UA), apolipoprotein A1(ApoA1), apolipoprotein B (ApoB), lipoprotein (a) (Lp(a)), hypertension, dyslipidemia, diabetes mellitus (DM). Body mass index (BMI) was calculated by dividing body weight (kg) by the square of height (m^2^). The venous blood samples were collected after overnight fasting before coronary angiography. Routine biochemical parameters including lipids, fasting blood glucose (FBG), and renal function. TyG index was defined as TyG = ln [fasting triglycerides (mg/dl) × fasting glucose (mg/dl)/2]. We used the baseline fasting triglycerides and FBG to calculate the TyG index^5,11^. Hypertension was defined as SBP ≥ 140 mmHg or DBP ≥ 90 mmHg, any use of the antihypertensive drug, or a self-reported history of hypertension. Diabetes was defined as FBG ≥ 7.0 mmol/L, any use of glucose lowering drugs, or a self-reported history of diabetes. Dyslipidemia was defined as any self-reported history or use of lipid-lowering drugs, total cholesterol (TC) ≥ 5.17 mmol/L. The concomitant treatment of this population was also documented. Definitions of CAD Risks were as follows: (1) Hypertension; (2) Diabetes mellitus; (3) Blood lipid abnormalities; (4) Obesity: BMI ≥ 28 kg/m^2^^12^. Our study population was non-smoking. We have defined hypertension, diabetes and dyslipidemia in the above statement.

### Angiographic analysis

Angiographic data were obtained from the cardiac catheterization laboratory records. Coronary angiography was performed by three dedicated interventionists by radial or femoral route depending on the operator’s discretion. The assessment of coronary artery was done by three senior-most cardiologists to maintain uniformity and to reduce the inter-observer variability. CAD was defined as coronary artery stenosis ≥ 50% in at least one major coronary artery.

### Statistical analysis

Statistical analyses were performed using R version 4.3.2. To assess whether variables followed a normal distribution, we utilized the Kolmogorov-Smirnov test. Continuous variables that conformed to a normal distribution were summarized as mean ± standard deviation, while those with non-normal distributions were reported as median (interquartile range). Categorical variables were presented as counts (percentages). To compare baseline characteristics between the CAD and non-CAD groups, we applied the unpaired Student’s t-test for continuous variables that were normally distributed. For continuous variables with non-normal distributions, the Mann-Whitney U test was used. Differences in proportions of categorical variables between the two groups were evaluated using the chi-square test; however, if any cell had an expected frequency of less than 5, Fisher’s exact test was employed.

To identify the most significant risk factors for coronary artery disease (CAD) in premenopausal women, we employed Least Absolute Shrinkage and Selection Operator (LASSO) regression. This was followed by multivariable logistic regression to further refine the selection of risk factors. Variables with a p-value < 0.01 in the multivariate analysis were considered statistically significant and were included in the construction of a nomogram. The nomogram was developed using the significant risk factors identified through the multivariable logistic regression model. Calibration of the nomogram was assessed by plotting a calibration curve, which provides a visual comparison between observed outcomes and predicted probabilities. The nomogram underwent bootstrap validation (1,000 bootstrap resamplings) to calculate the relatively corrected C-index. Additionally, Decision Curve Analysis (DCA) was conducted to evaluate the clinical utility of the nomogram, quantifying its net benefit across a range of threshold probabilities.

## Ethics approval and consent to participate

The study was performed according to the guidelines of the Helsinki Declaration and has been approved by the ethics committees at the First Hospital of Hebei Medical University, China. Since data were evaluated retrospectively, pseudonymously and were solely obtained for treatment purposes, a requirement of informed consent was waived by the Ethics Committee of the First Hospital of Hebei Medical University.

## Results

### Basic information

A total of 222 premenopausal women were included in the final analysis and were divided into a coronary artery disease (CAD) group and a non-coronary artery disease (CAD) group based on coronary angiography results. The coronary artery disease group consisted of 86 patients. The clinical characteristics of eligible premenopausal women are shown in Table 1. In premenopausal women, those with CAD are older than those without the condition. The median age of the CAD patients was 49 years (46–51 years), and the age of the no-CAD patients was 46 years (41–50 years). Moreover, in premenopausal women, those with CAD have a higher BMI (*p* < 0.029). Premenopausal women with higher systolic and diastolic blood pressure are more likely to develop coronary artery disease (*p* < 0.01). Among the laboratory indicators, compared with the no-CAD patients, the CAD patients were more likely to have lower total bilirubin, conjugated bilirubin, unconjugated bilirubin (*p* < 0.05), and have higer white blood cell (WBC), neutrophils (NEUT), aspartate transaminase (AST), alkaline phosphatase (ALP), fasting blood glucose (FBG), triglycerides (TG), lipoprotein (a) (Lp(a)) (*p* < 0.01). The TyG index in individuals with CAD is also higher than in those without CAD (*p* < 0.001). There were no significant differences in echocardiographic parameters between the two groups (*p* > 0.05). In postmenopausal women, those with hypertension, diabetes mellitus (DM), and hyperlipidemia have a greater risk of developing CAD (*p* < 0.001).

**Table 1:** Baseline characteristics of patients.

	Total population (*n* = 222)	NO-CAD *(n* = 136)	CAD *(n* = 86)	*p*-value
Age, years	48.00 [43.00, 51.00]	46.00 [41.00, 50.00]	49.00 [46.00, 51.00]	< 0.001
BMI, kg/m^2^	26.20 [23.50, 29.14]	25.67 [23.10, 28.62]	27.08 [24.16, 29.62]	0.029
Time, h	168.00 [72.00, 720.00]	192.00 [72.00, 720.00]	120.00 [30.00, 720.00]	0.011
*Vital signs*				
SBP, mmHg	129.00[118.00, 44.00]	125.00[114.00,142.25]	134.00[125.00,148.00]	0.002
DBP, mmHg	83.03 ± 11.68	81.29 ± 11.34	85.78 ± 11.74	0.005
RHR, beats per minute	76.00 [67.00, 84.00]	76.00 [68.75, 84.00]	76.00 [67.00, 83.75]	0.673
*Laboratory results*				
WBC, ×10^9^/L	5.85 [4.90, 7.30]	5.60 [4.80, 6.82]	6.15 [5.50, 7.95]	0.001
Neutrophils, ×10^9^/L	3.68 [3.00, 4.74]	3.53 [2.79, 4.39]	4.06 [3.23, 5.58]	0.001
RBC,×10^12^/L	4.33 ± 0.38	4.31 ± 0.38	4.35 ± 0.39	0.542
Hemoglobin, g/dL	128.00[120.00,136.00]	128.00[119.75,136.00]	128.00[120.00,136.75]	0.721
Platelet, ×10^9^/L	255.00[220.25,288.75]	245.50[215.25,287.25]	262.00[227.00,291.75]	0.227
AST, U/L	18.65 [15.90, 25.48]	17.60 [15.67, 23.92]	20.50 [16.58, 30.40]	0.004
ALT, U/L	16.30 [12.10, 26.60]	15.50 [11.83, 23.70]	18.15 [13.20, 28.32]	0.061
Albumin, g/L	41.01 ± 3.70	41.36 ± 3.50	40.46 ± 3.95	0.078
ALP, U/L	64.50 [54.00, 77.00]	60.00 [51.00, 69.25]	74.00 [61.25, 86.00]	< 0.001
Total bilirubin, g/L	11.40 [9.70, 14.88]	12.20 [10.00, 16.05]	11.00 [8.72, 13.75]	0.017
Direct bilirubin, g/L	2.20 [1.83, 3.00]	2.30 [1.90, 3.23]	2.10 [1.63, 2.68]	0.019
Indirect bilirubin, g/L	9.30 [7.73, 12.15]	9.65 [8.10, 12.53]	8.95 [6.90, 11.10]	0.021
Creatinine, µmol/L	53.75 [48.23, 58.88]	53.95 [48.48, 58.62]	53.35 [47.82, 60.60]	0.697
BUN, mmol/L	4.36 [3.64, 5.38]	4.17 [3.62, 5.35]	4.60 [3.69, 5.52]	0.229
UA, µmol/L	289.10[236.45,342.82]	288.60[227.85,338.98]	289.10[250.00,354.45]	0.282
FBG, mmol/L	5.00 [4.59, 5.69]	4.89 [4.53, 5.38]	5.24 [4.73, 7.22]	< 0.001
LDL-C, mmol/L	2.85 ± 0.65	2.81 ± 0.62	2.91 ± 0.69	0.292
HDL-C, mmol/L	1.14 [0.98, 1.29]	1.14 [1.01, 1.30]	1.10 [0.92, 1.26]	0.073
TG, mmol/L	1.23 [0.88, 1.70]	1.15 [0.83, 1.47]	1.40 [1.12, 1.85]	< 0.001
TC, mmol/L	4.58 [3.91, 5.11]	4.57 [3.90, 5.05]	4.61 [3.92, 5.26]	0.576
Non-HDL, mmol/L	3.42 [2.76, 3.94]	3.36 [2.72, 3.83]	3.50 [2.99, 4.04]	0.199
Lp(a), mg/L	147.00 [78.80, 318.78]	137.35 [69.05, 253.35]	177.20 [88.50, 467.58]	0.005
ApoA1, g/L	1.25 [1.15, 1.40]	1.26 [1.16, 1.40]	1.24 [1.09, 1.42]	0.288
ApoB, g/L	0.80 [0.64, 0.99]	0.79 [0.62, 0.97]	0.82 [0.66, 1.00]	0.203
TyG index	8.41 [7.99, 8.86]	8.26 [7.90, 8.57]	8.60 [8.18, 9.08]	< 0.001
*Echocardiography*				
LVEF, %	65.00 [62.00, 69.00]	65.50 [62.00, 69.25]	65.00 [62.00, 68.75]	0.529
LAD, mm	33.00 [30.00, 35.00]	32.50 [30.00, 34.00]	33.00 [30.00, 36.00]	0.245
RAD, mm	31.00 [28.00, 33.00]	30.50 [28.00, 33.00]	31.00 [29.00, 32.00]	0.875
RVD, mm	29.00 [27.25, 31.00]	29.00 [27.00, 31.00]	30.00 [28.00, 31.00]	0.428
LVEDD, mm	45.00 [43.00, 48.00]	45.00 [43.00, 48.00]	45.00 [42.25, 47.75]	0.865
LVESD, mm	29.00 [27.00, 31.00]	29.00 [27.00, 31.00]	29.00 [27.00, 31.00]	0.392
*Complications*				
Hypertension, n (%)	88 (39.6)	37 (27.2)	51 (59.3)	< 0.001
Diabetes mellitus, n (%)	39 (17.6)	9 (6.6)	30 (34.9)	< 0.001
Dyslipidemia, n (%)	119 (53.6)	59 (43.4)	60 (69.8)	< 0.001
Family history of CAD, n (%)	23 (10.4)	13 (9.6)	10 (11.6)	0.655

Dates are presented as mean ± SD, medians with inter quartile ranges or percentage. BMI body mass index, Time time from first symptoms to presentation, SBP systolic blood pressure, DBP diastolic blood pressure, RHR resting heart rate, *WBC* white blood cell count, RBC red blood cell count, AST glutamic oxaloacetic transaminase, ALT glutamic pyruvic transaminase, ALP alkaline phosphatase, BUN blood urea nitrogen, UA uric acid, FBG fasting blood glucose, LDL-C low-density lipoprotein cholesterol, HDL-C high-density lipoprotein cholesterol, TG triglycerides, TC total cholesterol, Non-HDL non-high density lipoprotein cholesterol, Lp(a) lipoprotein (a), ApoA1 apolipoprotein A1, ApoB apolipoprotein B, TyG triglyceride-glucose index, LVEF left ventricular ejection fraction, LAD left atrial diameter, RAD right atrial diameter, RVD right ventricular dimension, LVEDD left ventricular end-diastolic diameter, LVESD left ventricular end-systolic diameter.

### Screening for risk factors

**Fig. 2:**
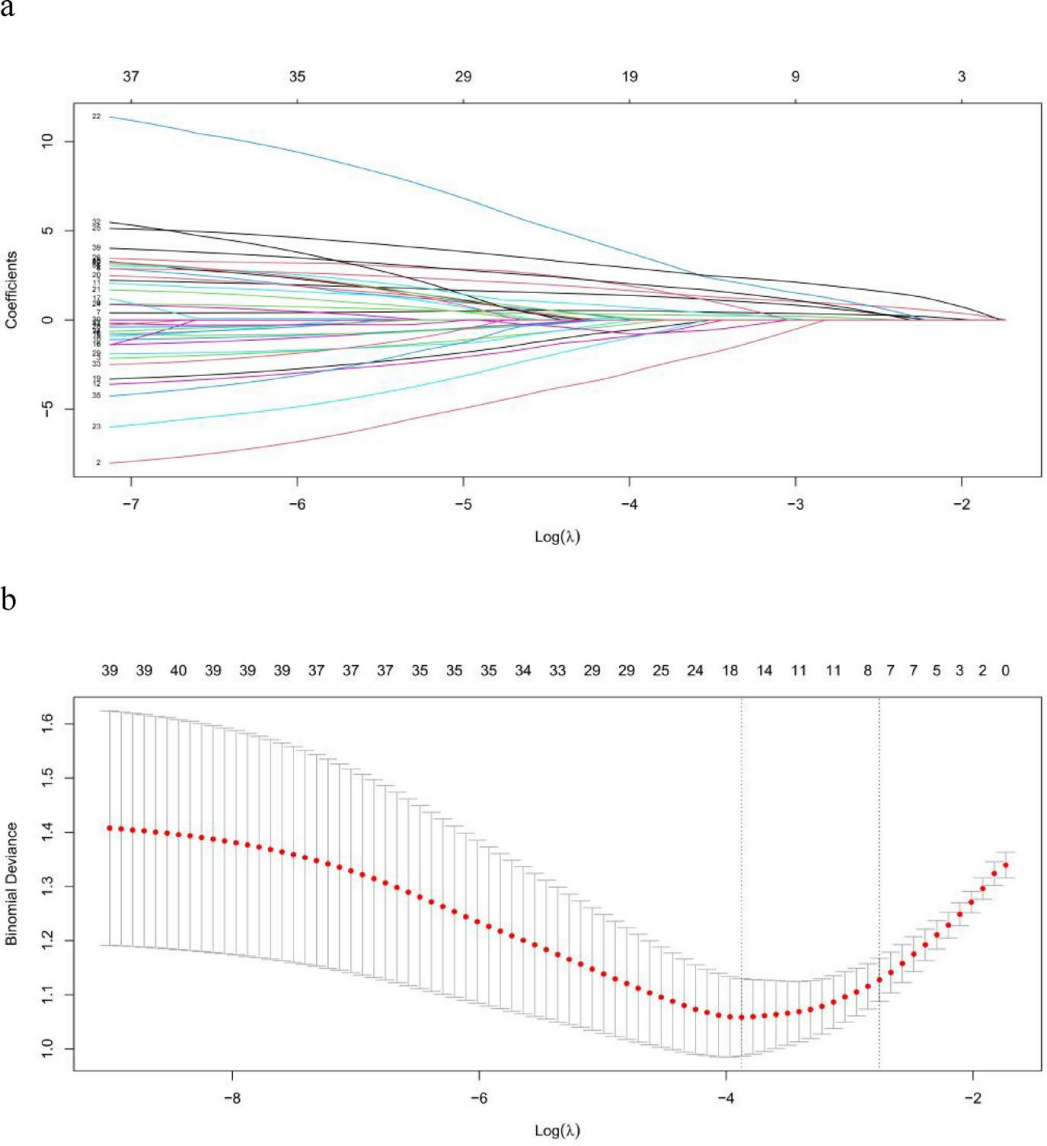
Use of the LASSO regression to select the variables.

41 predictive variables were introduced into the LASSO regression model. The LASSO regression screening employed 20-fold cross-validation to select the minimum penalty coefficient λ at the lowest point of the curve. Under this λ value, the model fitting effect was superior. On the right side of the curve, the penalty coefficient λ-se was chosen. Under this λ value, the fitting effect of the constructed model was also excellent. Meanwhile, the number of included equations was fewer and the model was simpler. Considering the practical application in clinical practice, we selected the λ-se on the right side as the screening criterion for the final equation, and ultimately included 7 predictive variables with nonzero coefficients (Fig. 2a, b).

These predictive variables comprise age, hypertension, diabetes mellitus(DM), hyperlipidemia, aspartate transaminase (AST), alkaline phosphatase (ALP), lipoprotein (a) (Lp(a)).

## The construction and validation of the nomogram

**Fig. 3:**
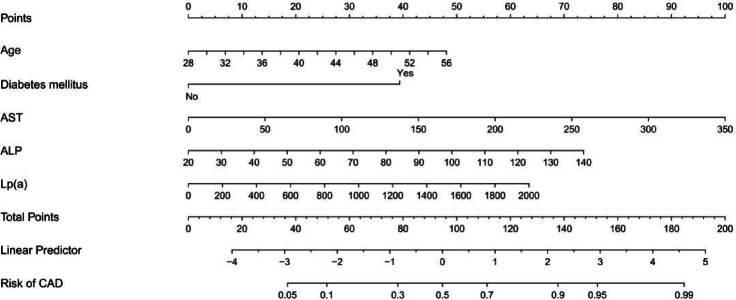
Nomogram for predicting CAD in premenopausal women.

**Fig. 4:**
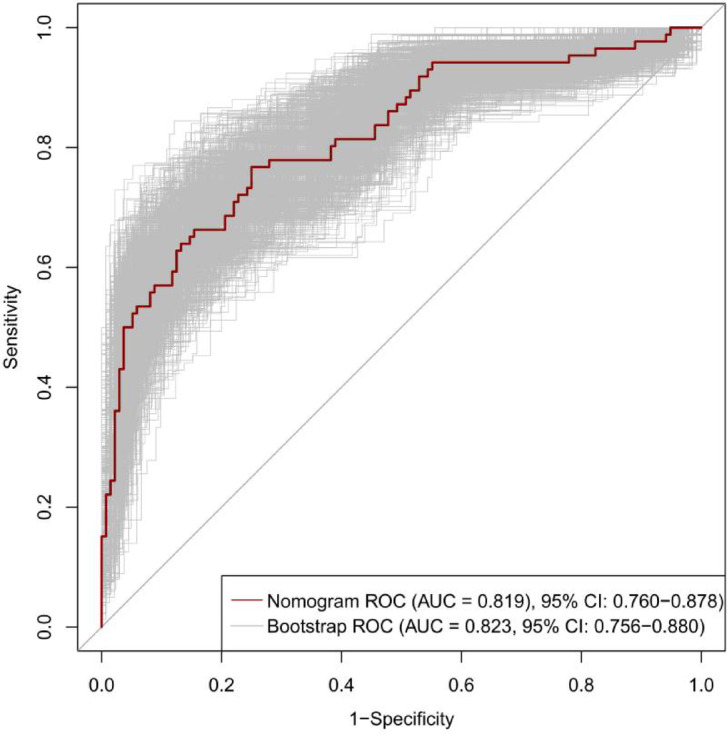
The ROC curve of the nomogram and Bootstrap Validation.

**Fig. 5:**
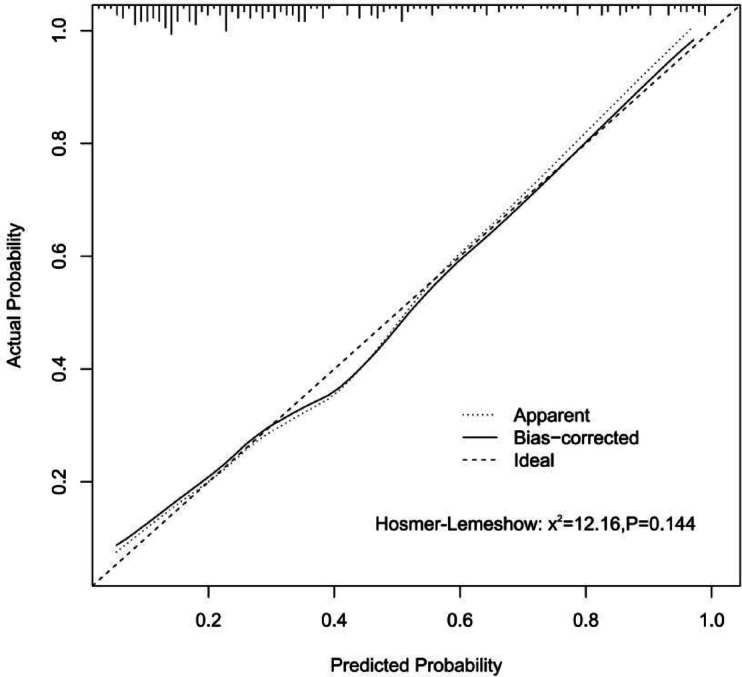
The calibration curve of the nomogram.

**Fig. 6:**
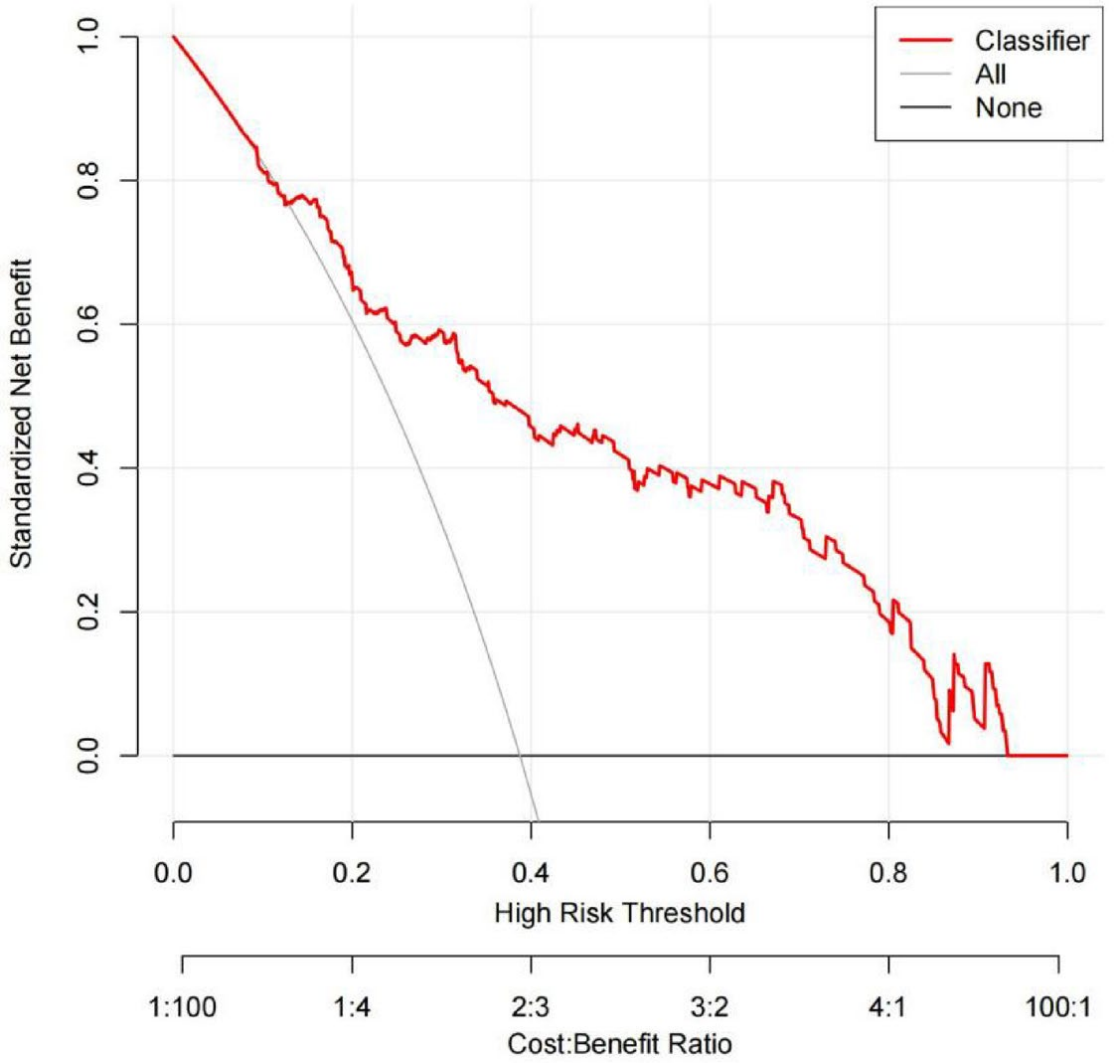
Decision curve analysis (DCA) of the nomogram.

The 7 predictive variables selected in the LASSO model were included in the multivariate logistic regression analysis. The study found that independent predictors of CAD in premenopausal women include age, diabetes mellitus(DM), aspartate transaminase (AST), alkaline phosphatase (ALP), lipoprotein (a) (Lp(a)) (*P* < 0.05, Table 2). Based on these findings, we have created a nomogram to predict the risk of coronary heart disease in premenopausal women (Fig. 3).The C-index of the predictive nomogram was 0.819 (95%CI: 0.760–0.878); the bootstrap validation yielded 0.823 (95%CI: 0.756–0.880), (Fig. 4), suggesting that the model had a good discriminatory capacity. Additionally, according to the Hosmer-Lemeshow test (x^2^ = 12.16, *P* = 0.144) and calibration curves, the predictive model demonstrated good performance (Fig. 5). The DCA curve demonstrates that within the 8%−93.3% threshold probability range, the nomogram maximizes clinical net benefit for study subjects, demonstrating clear superiority over both treat-all and treat-none strategies (Fig. 6).

**Table 2:** Multivariate logistic regression analysis of risk factors for CAD in premenopausal women.

variable	*p* value	Odds ratio (95% CI)
Age, years	0.0264	1.0798 (1.0090, 1.1556)
AST, U/L	0.0265	1.0130 (1.0015, 1.0246)
ALP, U/L	0.0028	1.0296 (1.0101, 1.0496)
Lp(a), mg/L	0.0216	1.0014 (1.0002, 1.0027)
Hypertension, n (%)	0.1878	1.6151 (0.7914, 3.2961)
Diabetes mellitus, n (%)	0.0003	5.5935 (2.1908, 14.2807)
Dyslipidemia, n (%)	0.1126	1.7598 (0.8755, 3.5374)

CAD coronary artery disease, AST glutamic oxaloacetic transaminase, ALP alkaline phosphatase, Lp(a) lipoprotein (a).

## Discussion

Premenopausal women have a relatively lower risk of developing CAD due to the cardiovascular protective effects of estrogen. These effects include improving lipid profiles, enhancing blood flow^10–12^, and providing benefits for both primary and secondary prevention of CAD^13–15^. However, this does not mean they are completely immune, and the incidence of CAD in this population has been increasing^8^. Therefore, early identification of high-risk patients and adequate preventive interventions are crucial for improving patient outcomes.This is the first predictive model to use common clinical indicators at admission to predict the occurrence of CAD in premenopausal women with suspected CAD.

Some of the risk factors identified in our study have been reported in previous research, such as age and DM, which are well-known common risk factors for CAD. Premenopausal women are generally younger, but those in the CAD group were older than those in the non-CAD group. The risk of CAD increases with age, possibly due to the accumulation of comorbidities^16^. DM increase the risk of coronary artery occlusion and myocardial ischemia by promoting intimal damage and atherosclerotic plaque formation. A meta-analysis of 64 cohort studies, including 858,507 patients, demonstrated that women with DM had a 2.82 times higher risk of CAD compared to those without DM, while the corresponding risk for men was 2.16 times higher^17,18^. Additionally, women with either type 1 or type 2 DM have a higher risk of cardiovascular disease compared to men^19^. However, women with DM are less likely to receive treatment compared to men.Therefore, timely detection and treatment of DM are crucial for reducing the risk of CAD in female patients.

Additionally, we found that elevated levels of Aspartate Transaminase (AST), Lipoprotein (a) (Lp(a)), and Alkaline Phosphatase (ALP) are independently associated with the occurrence of CAD in premenopausal women with suspected CAD, which has been rarely mentioned in previous studies.

Aspartate Transaminase (AST) is an enzyme widely present in various tissues, particularly in the liver, heart, muscles, and kidneys. While AST is primarily used as a marker of liver function, its role in heart disease, especially CAD, should not be overlooked. High levels of AST may indicate an increased risk of cardiovascular disease. A study has shown that blood AST levels are associated with the occurrence and progression of CAD^20^. Elevated AST levels may be linked to metabolic syndrome, which is a significant risk factor for CAD. Additionally, elevated AST levels may reflect systemic inflammatory responses, and chronic inflammation is a crucial factor in the development of CAD^21–24^. In our study, AST had the highest weight among all predictors. Higher AST levels were associated with a greater probability of CAD in premenopausal women.

Lipoprotein (a) (Lp(a)) is a large molecular complex found in plasma, involved in processes such as cholesterol transport, blood coagulation, fibrinolysis, and tissue repair^25^. In recent years, it has gained attention due to its role in the mechanisms of atherosclerosis, calcification, and thrombosis. Multiple large genetic studies and observational cohort studies have shown that blood Lp(a) levels are significantly associated with the risk of atherosclerotic cardiovascular disease (ASCVD), including CAD^26–29^. Lp(a) levels are primarily determined by genetic factors, leading to substantial variations across different races, regions, and individuals^30–32^. In premenopausal women, the protective effects of estrogen result in lower Lp(a) levels compared to postmenopausal women. However, high levels of Lp(a) are strongly associated with the incidence of CAD in this population. Further research is needed to clarify the specific mechanisms of Lp(a) in premenopausal women.

Alkaline phosphatase (ALP) is an enzyme that catalyzes the hydrolysis of inorganic pyrophosphate, a potent inhibitor of vascular calcification. It is widely expressed in various tissues throughout the body, with the highest activity in the liver, bones, and kidneys. Scientific research has shown that ALP acts as a regulator of vascular calcification by reducing the levels of inorganic pyrophosphate. Inorganic pyrophosphate provides protection to the vasculature, but ALP degrades its structure and activity, thereby promoting vascular calcification, which can lead to atherosclerosis^33–35^. In the process of atherosclerosis, inflammation plays a critical role. When inflammation occurs in the vasculature, endothelial cells are damaged, leading to endothelial dysfunction. The accumulation of activated inflammatory cells and the release of inflammatory factors accelerate the progression of atherosclerosis. Research has shown a correlation between ALP and C-reactive protein (CRP)^36^, indicating that serum ALP is associated with inflammation. ALP may contribute to the atherosclerotic process through inflammatory pathways.A meta-analysis of four prospective studies demonstrated that each standard deviation increase in baseline ALP levels was associated with an 8% increase in the risk of CAD^37^. In our study, we found that high levels of ALP remain an independent risk factor for CAD in premenopausal women, a specific subgroup.

Previous studies have proposed CAD prediction scores, with the Duke score and GRACE score being commonly used in clinical practice. However, the GRACE score is primarily designed to assess the risk in patients with acute coronary syndromes (ACS), while the Duke score is based on European and American populations, whose disease characteristics differ from those of Chinese populations. Additionally, these scores have not been specifically tailored for premenopausal women^38–40^. One advantage of our new nomogram is its simplicity and the use of common data available upon admission. Furthermore, the nomogram is easy to calculate and has good clinical applicability. Finally, the calibration curve of the prediction model is excellent, and decision curve analysis shows that the model offers significant net benefit in clinical applications.

## Study limitations

This study has several limitations that need to be considered. First, this is a single-center retrospective analysis, and selection bias is inevitable. Second, the sample size is relatively small, which may affect the reliability of the results. Third, our study population is limited to Han Chinese individuals, making it difficult to generalize the current prediction model to all countries and ethnic groups. The applicability of this model to premenopausal women in other regions and countries remains unknown and requires testing in larger and more diverse premenopausal populations.

## Conclusion

Finally, based on our study results, a nomogram incorporating six predictive factors was developed. The nomogram has an AUROC of 0.838 and demonstrates excellent performance in discrimination, calibration, and clinical application, aiding doctors in the initial assessment of CAD risk in premenopausal women with suspected CAD.

## Data Availability

Due to the small amount of data in this study and the fact that we will collect further data for subsequent studies, the datasets generated and/or analysed during the current study are not publicly available but are available from the corresponding author on reasonable request.

## Abbreviations

CAD: Coronary artery disease
LASSO: Least absolute shrinkage and selection operator
OR: Odds ratio
ROC: Receiver operating characteristic
AUROC: Area under the receiver operating characteristic curve
DCA: Decision curve analysis
BMI: Body mass index
Time: Time from first symptoms to presentation
SBP: Systolic blood pressure
DBP: Diastolic blood pressure
RHR: Resting heart rate
WBC: White blood cell count
RBC: Red blood cell count
AST: Glutamic oxaloacetic transaminase
ALT: Glutamic pyruvic transaminase
ALP: Alkaline phosphatase
BUN: Blood urea nitrogen
UA: Uric acid
FBG: Fasting blood glucose
LDL-C: Low-density lipoprotein cholesterol
HDL-C: High-density lipoprotein cholesterol
TG: Triglycerides
TC: Total cholesterol
Non-HDL: Non-high density lipoprotein cholesterol
Lp(a): Lipoprotein (a)
ApoA1: Apolipoprotein A1
ApoB: Apolipoprotein B
TyG: Triglyceride-glucose index
LVEF: Left ventricular ejection fraction
LAD: Left atrial diameter
RAD: Right atrial diameter
RVD: Right ventricular dimension
LVEDD: Left ventricular end-diastolic diameter
LVESD: Left ventricular end-systolic diameter
